# Correlative Study on the Relationship between the Expression of m6a-Related Genes and the Prognosis and Immunotherapy of Soft Tissue Sarcoma

**DOI:** 10.1155/2022/5439023

**Published:** 2022-07-13

**Authors:** Yue Qiu, Jia Li, Jun Yao, Jinzhi Meng, Xing Huang, Xifan Zheng, Zhenpei Wen, Junpu Huang, Hongtao Wang

**Affiliations:** ^1^The First Affiliated Hospital of Guangxi Medical University, Nanning 530021, China; ^2^Department of Pathology, The First Affiliated Hospital of Guangxi Medical University, Nanning 530021, China

## Abstract

**Background:**

Soft tissue sarcomas (STS) are rare malignancies arising from mesenchymal tissue and interlacing ectodermal nerve tissue. Immunotherapy plays an important role in the prognosis and survival of STS patients. However, there is insufficient evidence to confirm the prognostic value of m6A-related genes and to evaluate the efficacy of immunotherapy for STS.

**Methods:**

We analyzed 23 m6A regulators from STS samples using R software and defined the modification patterns for three STS m6A regulators. Then, we constructed the m6A scores and divided the samples into high and low subgroups. Finally, we used data from the GEO database to verify the results.

**Results:**

We found that the m6A clusters differed in the overall survival (OS), progression-free survival (PFS), and immune infiltration rate. Additionally, the m6A score was positively correlated with the contents of activated B cells, activated dendritic cells, CD56 bright natural killer cells, helper T cells, and regulatory T cells. The group with a higher m6A score also presented higher OS and PFS rates. Regarding immunotherapy, STS patients with a high m6A score presented better results. Consistently, we found similar results in another dataset with patients that received anti-PD-1/PD-L1 therapy.

**Conclusion:**

Our current results indicated that the m6A score can be used to assess the survival rate of STS patients and guide immunotherapy and predict its effects. The analysis of different m6A patterns of STS samples contributed to the understanding of the diversity and complexity of the tumor microenvironment (TME) and provided new ideas for the clinical development of personalized immunotherapy and prediction of the prognosis of STS patients.

## 1. Introduction

Soft tissue sarcoma (STS) is a relatively rare type of malignant tumor compared to other tumors. It is more common in children, accounting for about 7-15% of malignant tumors in this population and about 1% in adults [1]. STS is a malignant tumor derived from the intertwining of mesenchymal and ectodermal nerve tissues and is composed of more than 50 different tissue subtypes with different pathological and clinical characteristics [2]. Although STS can occur in various parts of the body, it mainly occurs in the limbs, accounting for about 60% of the total [3]. The 5-year overall survival rate of STS patients is about 50% [4], and the median survival time is between 39.0 and 82.7 months [5, 6]. Preoperative or postoperative radiotherapy or chemotherapy, coupled with improvements in surgical methods, has improved the prognosis of patients with local diseases. Despite these advances, about 50% of patients will relapse, often with distant metastases [7]. In summary, STS lacks an efficient treatment plan, and how to control its development and distant metastasis remains a difficult problem. Therefore, finding prognostic indicators for STS patients is of great significance for the understanding of the disease and the treatment and evaluation of the prognosis of STS patients.

Furthermore, RNA modifications are chemical changes in the mature RNA chain of nucleotides at the posttranscriptional regulatory level [8]. Until now, more than 150 RNA modifications have been identified, including N6-methyladenosine (m6A), N1-methyladenosine (m1A), and 5-methylcytosine [9, 10]. Among the many RNA modifications, m6A is the most common and main type of internal modification, accounting for 0.1-0.4% of the total adenosine residues [11–13]. As a reversible modification behavior, the regulation of m6A methylation is mainly composed of three main regulators: methyltransferases, demethylases, and m6A-binding proteins [14]. The main regulatory genes of methyltransferases (also known as “writers”) are METL3, WTAP0.16, and METL14, and their main role is to induce methylation modification of the m6A mRNA base [15]. The base removal process of methylation modification is mediated by genes such as FTO and ALKBH5 and is also defined as “erasers” and comprehends the main function of demethylases [16]. The m6A-binding proteins are known as “readers” and are regulated by YTHDF1/2/3, YTHDC1/2, hnRNPA2B1, LRPPRC, and FMR1 regulatory genes to initiate downstream regulatory pathways by recognizing potential m6A-modified bases [16]. According to current research, m6A modification plays a vital role in almost all life activities of the human body and human diseases [17]. For example, m6A modifications are indispensable in spermatogenesis [18], tissue development [16], T cell homeostasis [19], DNA damage [20], heat shock response [21], and other processes. However, the abnormal modification of the m6A gene and the abnormal expression of m6A regulatory proteins often lead to a variety of diseases, such as acute myeloid leukemia [22], breast cancer [23], hepatocellular carcinoma [24], neurological diseases [24], and autoimmune diseases [25]. Disease causes are related to the disorder of various biological processes, such as the imbalance of cell death and proliferation, the malignant progression of tumors, cell development, abnormal immune regulation, and impaired self-renewal ability [26–28].

For a long time, it was believed that tumor progression was a process involving only genetic and epigenetic changes in tumor cells. However, according to current research, the tumor microenvironment (TME) on which tumor cells depend for growth and survival also plays an indispensable role in the occurrence and development of malignant tumors. The TME participates in immune escape, tumor progression, and response to immunotherapy. Previous studies have shown that the regulatory gene FTO of m6A demethylase has an essential influence on the response of skin melanoma patients to anti-PD-1 immunotherapy [29]. Additionally, Shi et al. have reported that the low expression of the m6A-binding protein factor YTHDF1 in the body can cause resistance to cisplatin therapy in non-small-cell carcinoma (NSCLC) patients, and the therapeutic effect is poor [30]. The abnormal regulation of YTHDF1 can also lead to the proliferation of non-small-cell carcinoma cancer cells and accelerate disease progression [30].

Due to the complexity and heterogeneity of the TME, there are few studies regarding its role in STS. Hence, this study is aimed at comprehensively analyzing the heterogeneity and complexity of m6A regulatory factors in STS patients and its TME landscape to find different tumor immunophenotypes and new STS biological markers and improve the guidance and the ability to predict the response of immunotherapy and find new therapeutic targets for STS. Herein, we integrated the genome information of 180 STS patients (including TCGA and GEO databases). We analyzed three different m6A modification patterns and clarified the important role of m6A modifications in the TME of STS. Additionally, we established a set of m6A-related scoring systems to quantify the m6A modification patterns of individual patients, for predicting the prognosis of STS patients and the efficacy of immunotherapy.

## 2. Methods

### 2.1. Data Acquisition and Processing

First, 120 STS-related sample data were retrieved from The Cancer Genome Atlas (TCGA) database (https://portal.gdc.cancer.gov/). The data information includes transcriptome RNA sequence (FPKM value), copy number variation (CNV), single-nucleotide polymorphisms, and clinical data of STS patients (Table 1). Due to the lack of normal sample data in the TCGA database, we also downloaded the RNA sequences of 86 normal tissue samples from the University of California Santa Cruz Xena database (https://xena.ucsc.edu/). Additionally, the GSE17118 dataset for joint analysis with TCGA data was downloaded from the GEO database (https://www.ncbi.nlm.nih.gov/geo/), which contains 60 STS samples and related clinical information (Table 2). First, we used the “limma” R package to convert the FPKM values of the STS data downloaded from TCGA into TPM values. Then, we used this package to calibrate and integrate the 86 normal samples obtained by UCSC and TCGA's STS samples. The normal samples were placed in the front, and the STS samples were placed in the back. Finally, we sorted and standardized the data downloaded from the GEO database. Next, we used the “limma” and “sva” packages to combine the 120 STS samples from TCGA database and 60 samples from the GEO database for subsequent analysis. Additionally, the GSE17618 and GSE17674 datasets were downloaded from GEO, including sample information from 63 STS to verify the overall survival (OS) of the model. The GSE30929 cohort included the information from 140 STS patients and was used to validate the progression-free survival (PFS) in the model.

### 2.2. Clustering Analysis of the 23 m6A Regulators

We searched the PubMed database and retrieved a total of 23 m6A regulators: METTL3, METTL14, METTL16, WTAP, VIRMA, ZC3H13, RBM15, RBM15B, YTHDC1, YTHDC2, YTHDF1, YTHDF2, YTHDF3, HNRNPC, FMR1, and L RPPRC, HNRNPA2B1, IGFBP1, IGFBP2, IGFBP3, RBMX, FTO, and ALKBH. The “ConsensuClusterPlus” R package was used to cluster STS patient data. According to the expression of the 23 m6A regulators, STS patients were classified to determine different m6A modification patterns. This step was repeated 1000 times to ensure stable classification [31].

### 2.3. Gene Set Variation Analysis (GSVA) and Evaluation of Relative Abundance of Infiltrating Immune Cells

The GSVA is a nonparametric and unsupervised algorithm mainly used to estimate the variation characteristics of pathways and biological process activities in the expression dataset [32]. Relevant gene sets were downloaded from the MSigDB database (gene set: c2.cp.kegg.v7.4.symbols.gmt), and the GSVA was performed using the “GSVA” R package (*p* < 0.05). Then, a single-sample gene set enrichment analysis (ssGSEA) was used to evaluate the differences in the abundance of infiltrated immune cells in TME under different m6A modification patterns in STS patients.

### 2.4. CIBERSORT Immune Infiltration Analysis

CIBERSORT was first published in *Nature Methods* in 2015 and is the most frequently cited tool for estimating and analyzing immune cell infiltration. CIBERSORT is a tool for deconvolution of expression matrices of human immune cell subtypes based on linear support vector regression. For on-chip expression matrix and sequencing expression matrix, deconvolution analysis for unknown mixtures and expression matrix containing similar cell types is superior to other methods. CIBERSORT was used to analyze immune infiltration in STS patients.

### 2.5. Identification of Differential Genes between Different m6A Modification Patterns

To identify the differences of m6A-related genes among different m6A modification patterns, we used the “limma” R package. We obtained a total of 121 m6A-related differential genes (adj. *p* value < 0.05).

### 2.6. Prognostic-Related Genes and Unsupervised Cluster Analysis

A univariate Cox regression pattern was used to analyze the differential genes related to the prognosis of STS patients. Four genes that were significant to the prognosis of STS were obtained for further analysis. According to prognostic genes, we used unsupervised clustering analysis to divide STS patients into three different gene subgroups: geneClusterA, geneClusterB, and geneClusterC.

### 2.7. Construction of the m6A Scoring System for STS Patients (m6A Score)

Through the above analysis, we obtained several m6A modification patterns of STS. However, these modification patterns were detected considering all STS patients, and there is no specific quantitative index specifying these individuals. Thus, to evaluate the m6A modification pattern specific to each STS patient, we established a special scoring system: the “m6A score”. Previously, we obtained genes that are meaningful to the prognosis of STS patients. Then, using principal component analysis (PCA), we scored each STS patient based on their expression of prognostic-related genes. The specific steps were as follows: first, we defined the gene features of m6A as A and B. A represents a positive correlation with DEGs, and B represents a negative correlation with DEGs. Then, we used PCA to reduce the dimensionality and finally obtained PC1 and PC2, which represent the positive correlation between the m6A gene feature score and the negative correlation gene score. The m6A score can be calculated according to the following formula:

(1) $$\begin{array}{c} \text{m}6\text{A score}=\sum \text{P}\text{C}1a+\sum \text{P}\text{C}2a, \end{array}$$

where “*a*” represents the expression of genes related to the m6A phenotype.

After calculating the m6A score of each patient through the above steps, we divided the STS patients into a high m6A score group and a low m6A score group for subsequent analysis.

### 2.8. Correlation Analysis of STS Clinical Features

First, we retrieved the somatic mutation data of STS patients from TCGA database to analyze the types and characteristics of cell mutations. The R software was used to analyze the patients with high and low m6A scores, the “maftools” package was used to display the mutations of patients with high and low m6A subtypes in the cohort, and the waterfall chart of the first 20 mutant genes was drawn. The “survival” R package was used to analyze the survival of the high and low mutation groups. To further explore the survival rate difference in m6A scores among different clinical characteristics, the survival analysis was carried out for different age groups (>65 years or ≤65 years) and genders (male or female) in the high and low m6A score groups.

### 2.9. Immunotherapy Value of the m6A Score

Due to the lack of efficient treatments for STS and the success in the field of immunotherapy for malignant tumors such as melanoma, non-small-cell lung cancer, and prostate cancer, STS immunotherapy has attracted increasing attention. One of the most successful treatment strategies is immune checkpoint inhibitor (ICIS) combination therapy [7]. First, we checked the relevant literature to obtain immune checkpoint blockade- (ICB-) related genes (PDCD1, CD274, and CTLA-4) [7] and then used the “limma” R package to analyze them in the high and the low m6A score groups. The difference between these genes indicated that the m6A high group might be more suitable for receiving anti-PD-1/PD-L1 or anti-CTLA4 immunotherapy. Finally, to verify the value of the m6A score in predicting immunotherapy response, we retrieved the independent cohort GSE78220 receiving anti-PD-1/PD-L1 treatment from the GEO database and verified the results obtained from the above analysis.

### 2.10. Statistical Analyses

The Wilcox test was used for comparisons between two groups, and the Kruskal-Wallis test was used for comparisons between two or more groups. The “SurvCutpoint” function was used to dichotomize m6A scores and divide patients into the high and low m6A score groups. The survival analysis curve was drawn by the Kaplan-Meier method. The survival difference was analyzed using the log-rank test. All data analyses in this study were performed in R software (version 4.1.1) and Perl (version 5.3.0). A *p* < 0.05 was defined as statistically significant.

## 3. Results

### 3.1. Genetic Variation Landscape of m6A Regulatory Genes in STS

**Figure 1:**
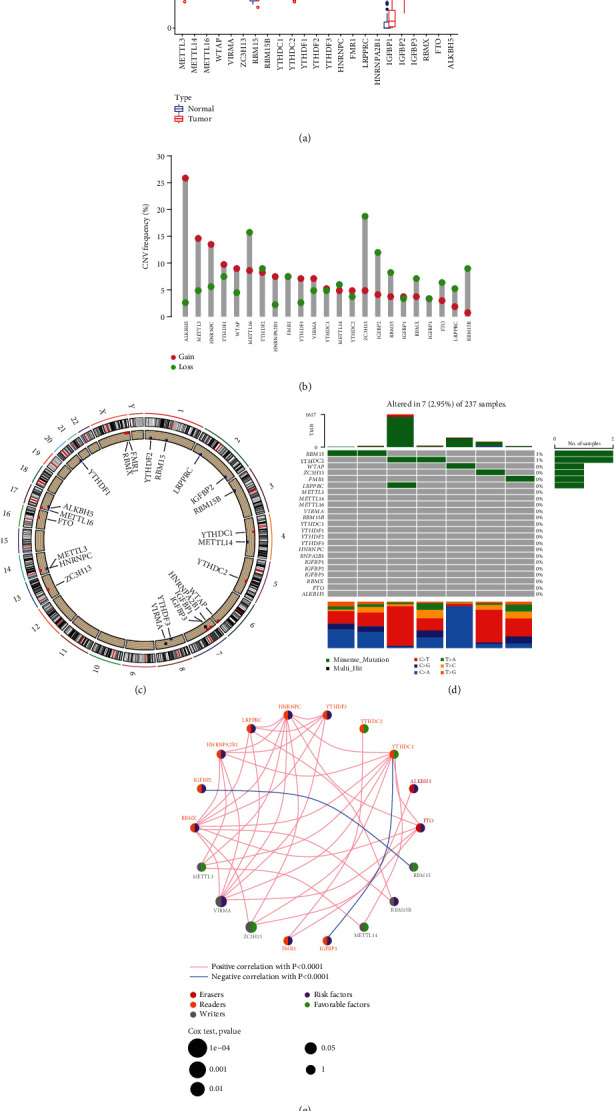
Genetic and expression variation landscape associated with the 23 m6A regulatory factors in soft tissue sarcoma (STS). (a) Comparison of the expression levels of the 23 m6A regulatory factors in normal soft tissue and STS. Blue represents normal soft tissue, and red represents STS tissue; blue dots and red dots represent abnormal values of normal and tumor tissues, respectively, and asterisks represent ^∗^*p* < 0.05, ^∗∗^*p* < 0.01, and ^∗^*p* < 0.001. (b) The CNV frequency diagram of 23 m6A regulatory factors in STS. Red and green represent gains and losses, respectively. (c) The CNV of 23 m6A regulatory factors of human chromosomes. Red indicates that the copy number has increased more than it is lost, and blue is the opposite. (d) Frequency waterfall diagram of CNV and somatic mutation of the 23 m6A genes in STS. (e) Prognostic coexpression network of m6A genes. The red, orange, and gray circles correspond to “rubber,” “reader,” and “writer,” respectively. The size of the circle represents the prognosis of STS patients.

**Figure 2:**
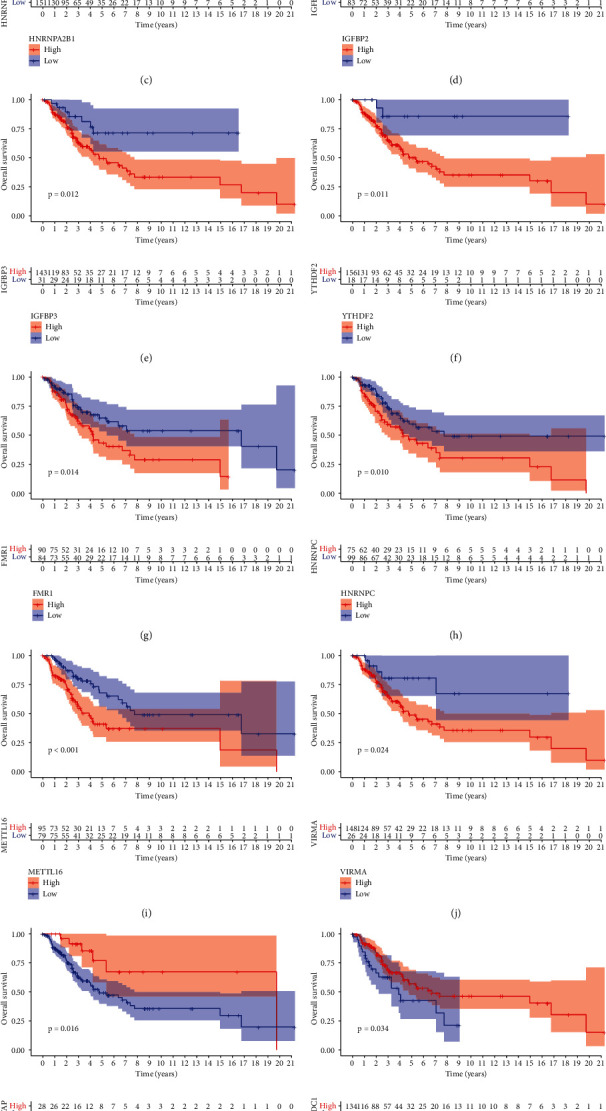
Survival analysis of 14 m6A regulators related to the prognosis of STS patients.

Herein, we identified 23 m6A regulators. Due to the lack of normal samples in the STS dataset from TCGA database, we searched for the RNA sequence data of 86 corresponding normal tissue samples from the UCSC Xena database. Then, we used R software to merge the two datasets after proofreading and to evaluate the differences in the expression of the 23 m6A regulators between STS and normal tissues (Figure 1(a)). Subsequently, to study the CNV of the m6A regulators in STS, we used R software to cross the 23 m6A regulators with the CNV data. The results showed that, in STS patients, the frequency of m6A gene copy number acquisition was low and the gene copy loss frequency of ZC3H13, IGFBP3, and RBM15B was significantly higher than the obtained frequency (Figure 1(b)). To study the performance of the 23 m6A regulators on each chromosome, we used the “RCircos” package (Figure 1(c)). Next, we summarized the CNV frequency and somatic mutation of the 23 m6A regulons in STS. The results showed that, out of 237 STS samples, 10 samples had genetic mutations (mutation frequency of 4.22%). The four m6A regulators, ZC3H13, FMR1, YTHDC2, and RBM15, presented the highest mutation frequency (Figure 1(d)). Next, we mapped the prognostic coexpression network of the m6A gene. Except for IGFBP2 and RBM15, and YTHDC1 and IGFBP1, the overall interaction between prognostic m6A genes was positively correlated, among which VIRMA and ZC3H13 were significantly correlated with the prognosis of STS patients (Figure 1(e)). Besides, we screened out 14 m6A regulators related to STS survival and prognosis through Cox and KM analyses: TME21, ALKBH5, FMR1, FTO, HNRNPA2B1, HNRNPC, IGFBP2, IGFBP3, METTL16, VIRMA, WTAP, YTHDC1, YTHDF2, YTHDF3, and ZC3H13 (Table 3). Finally, we performed a survival analysis with these prognostic-related m6A regulators (Figure 2).

### 3.2. Methylation Modification Patterns of the 23 m6A Regulators

**Figure 3:**
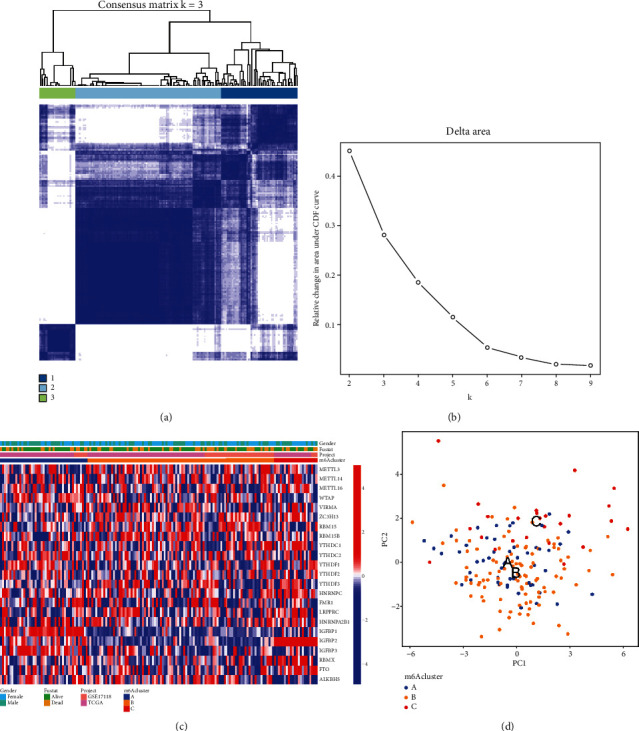
Cluster analysis based on the 23 m6A regulators in STS. (a) Consensus clustering subgroups (*K* = 3). (b) Relative change of the area under the CDF curve. (c) Heat maps of the m6A galaxy cluster under three correction modes. (d) Principal component analysis (PCA) under different m6A methylation modification modes.

To investigate the roles and mechanisms of m6A modulators in STS, we performed a consensus clustering analysis of the 23 m6A modulators using the “ConsensusClusterPlus” R package. When *K* = 3, the cluster was closely related, the intersection outside the cluster was the smallest, and the area under the curve presented the smallest changes (Figures 3(a) and 3(b)). Hence, we chose to divide the m6A cluster into three groups for the next analysis. We identified three different m6A modification patterns and drew the heat maps of the m6A clusters under the three modification patterns (Figure 3(c)). The 23 m6A regulators were highly expressed in the C cluster. Then, PCA was used to observe the expression changes in the three molecular subgroups. We observed that the level of m6A regulators can indeed distinguish STS patients into three different subgroups (Figure 3(d)).

### 3.3. GSVA, ssGSEA, and CIBERSORT between m6A Molecular Clusters with Three Different Modification Patterns

**Figure 4:**
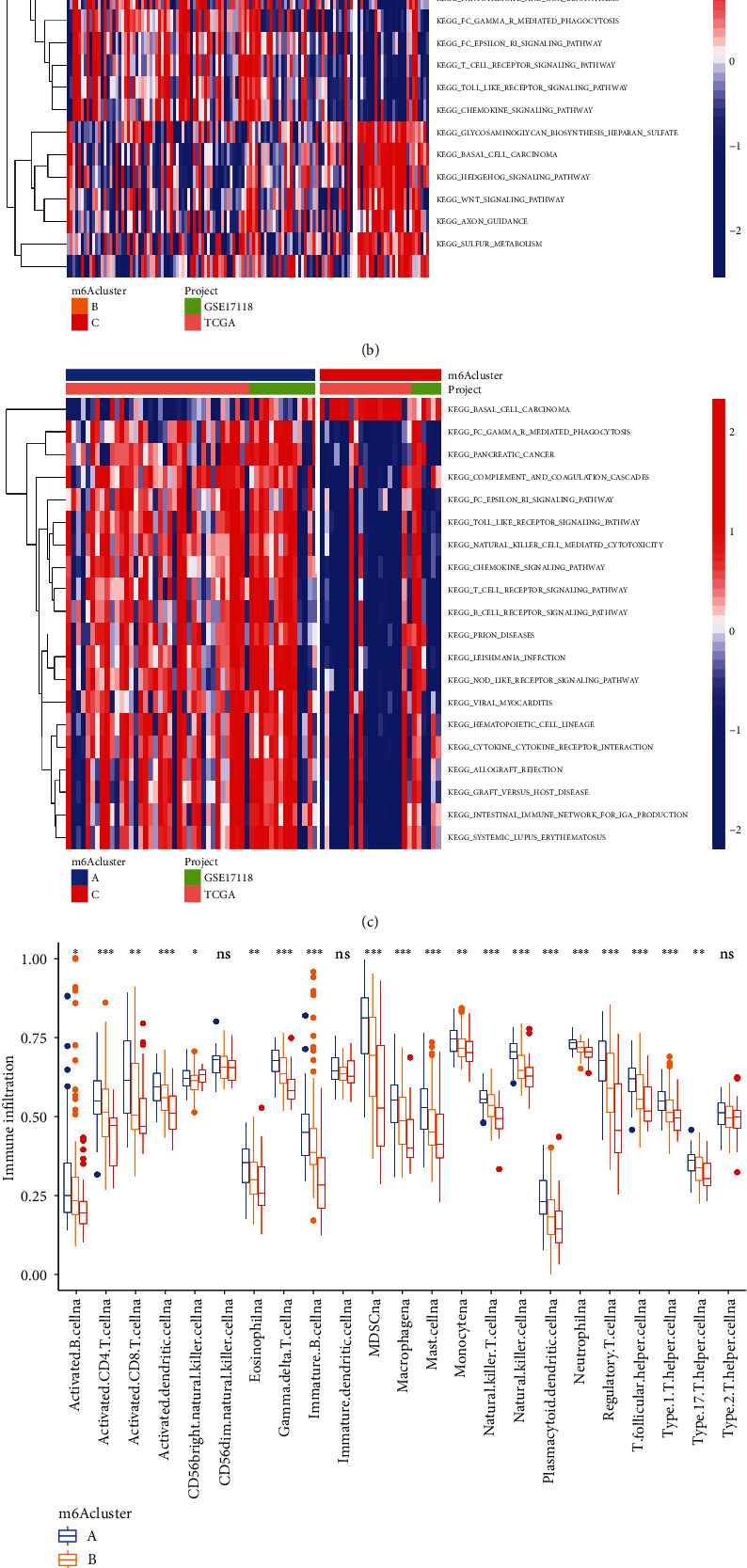
Comparison of GSVA and ssGSEA between 3 different m6A modification modes. (a) GSVA between A and B clusters. (b) GSVA between B and C subclusters. (c) GSVA between A and C subclusters. (d) Immune cell infiltration of different m6A modification patterns in the STS immune microenvironment. ^∗∗∗^*p* < 0.001, ^∗∗^*p* < 0.01, and ^∗^*p* < 0.05. (e) Immune cell infiltration of different m6A modification patterns in the STS immune microenvironment (CIBERSORT). ^∗∗∗^*p* < 0.001, ^∗∗^*p* < 0.01, and ^∗^*p* < 0.05, respectively.

Further, to have a more in-depth and detailed understanding of the biological action pathways between the m6A clusters under the three different modification patterns, we used GSVA. The comparison between the A and B groups showed that the expressions of primary bile acid biosynthesis, complement and aggregation cascade, leukocyte transendothelial migration, and other pathways in the A group were higher than those in the B group (Figure 4(a)). In the comparison between the B and C groups, the B group was mainly concentrated in acute myeloid leukemia, chronic myelogenous leukemia, and pancreatic cancer. Meanwhile, the C group was mainly concentrated in glycosaminoglycan biosynthesis heparin sulfate, ASAL cell carcinoma, and the HEDGEHOG signaling pathway (Figure 4(b)). In the pairwise comparison between the A and C groups, the biological regulation pathways of the A group were mainly concentrated in pancreatic cancer, complement and aggregation cascade, spot-like receptor signaling pathway, and T cell receptor signaling pathway, while the C group was only significantly enriched in the basal cell carcinoma pathway (Figure 4(c)). Then, to evaluate the relative abundance of TME-infiltrating immune cells in the three modification patterns, we performed a ssGSEA. The results suggested that the abundance of most infiltrating immune cells was significantly different between the three groups (*p* < 0.05) (Figure 4(d)). Additionally, the CIBERSORT immune infiltration analysis revealed significant differences in the abundance of macrophages and naive CT 4T cells between the three groups (Figure 4(e)), suggesting that the expression levels of m6A modulators can be used in the clinical evaluation of STS patients and direct immunotherapy.

### 3.4. Analysis of Differential Genes in the Three m6A Modification Patterns

**Figure 5:**
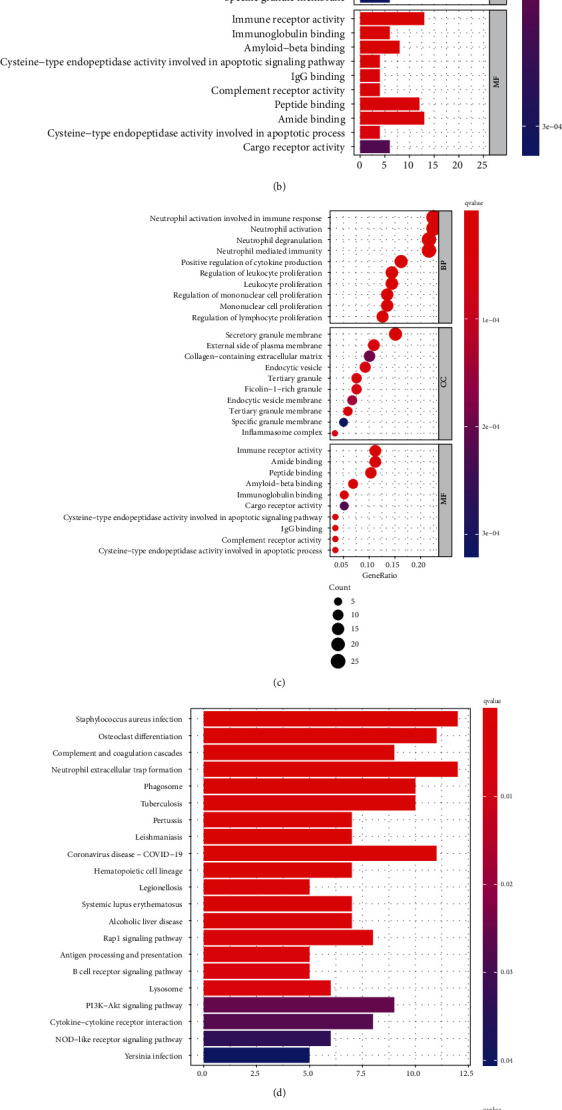
Identification and functional annotation of differential genes. (a) Venn diagram of cross genes between different m6A subclusters in STS. (b, c) GO function enrichment analysis of differential genes. (d, e) KEGG pathway enrichment analysis of differential genes.

Moreover, to study the potential biological behavior of each m6A modification pattern, we used the “limma” R package and obtained 121 m6A phenotypic-related differentially expressed genes (DEGs) (adjusted *p* value < 0.05). Then, we performed GO functional and KEGG enrichment analyses for these genes (Figure 5(a)). The GO enrichment analysis function can be divided into biological processes (BP), cell components (CC), and molecular functions (MF). In BP, these genes were mainly enriched in the immune response of neutrophil activation and neutrophil degranulation and neutrophil-mediated immune response. In CC, these genes were mainly enriched in the secretory granular membrane, tertiary granular membrane, and the outer side of the plasma membrane. In MF, genes were mainly enriched in the activity of immune receptors (Figure 5(b)). Overall, the results of GO functional enrichment analysis indicated that these 121 genes might be involved in the activation of immune cells. Consistently, in the KEGG enrichment analysis, these genes were mainly enriched in Staphylococcus aureus infection, complement and aggregation cascades, and formation of extracellular traps in neutrophils (Figure 5(c)). The results of GO and KEGG enrichment analysis demonstrated that these genes are significantly related to m6A modification and immunity, which reinforced the important role of m6A modification in the immune regulation of the TME.

### 3.5. Construction of m6A-Modified Genome Phenotypes

**Figure 6:**
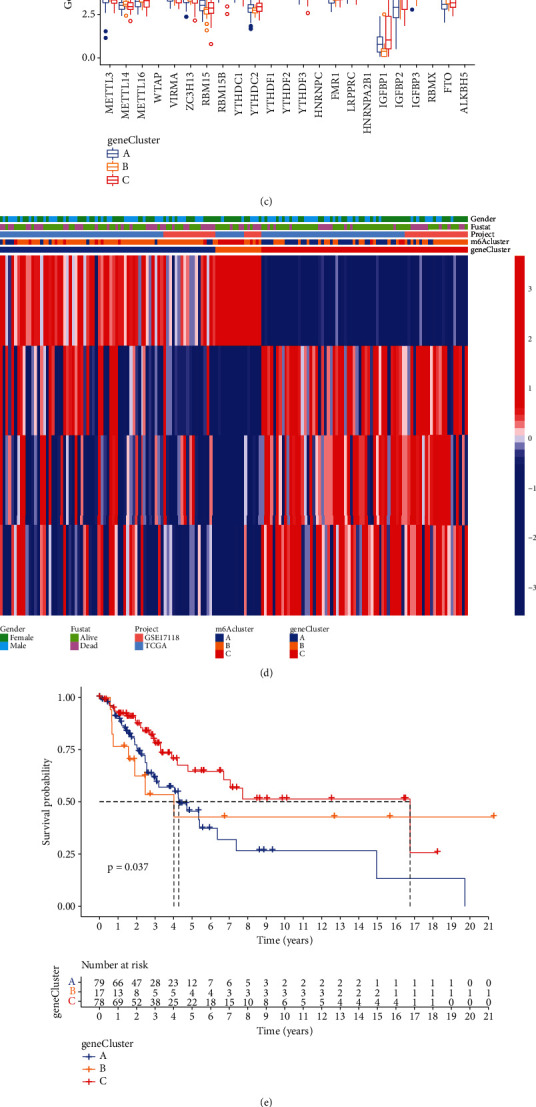
Construction of m6A-modified genome phenotype. (a) Gene consensus cluster subgroup when *K* = 3. (b) Relative change of the area under the CDF curve. (c) Block diagram of the expression differences of the 23 m6A regulators among three different gene clusters. (d) Different gene heat maps and different clinical characteristics of three groups of different gene clusters. (e) Kaplan-Meier survival analysis of STS patients with three different gene clusters (OS). (f) Kaplan-Meier survival analysis of STS patients with three different gene clusters (PFS).

To better understand the regulatory mechanisms of these m6A phenotype-related genes, we adopted a method similar to clustering of m6A modification patterns, called unsupervised cluster analysis. We divided STS patients into three different m6A-modified genome manifestations: geneClusterA, geneClusterB, and geneClusterC (Figures 6(a) and 6(b)). Next, we drew a block diagram using R software (Figure 6(c)) and showed that the expression levels of m6A-regulated genes were significantly different in these three groups, consistent with the results of the different m6A modification patterns above. Additionally, WTAP, IGFBP1, IGFBP3, and ALKBH5 presented the highest expression levels in geneClusterC, while METL3, YTHDC1, HNRNPC, LRPPRC, HNRNPA2B1, and RBMX presented the lowest expression levels in this cluster. The heat maps based on the different clinical characteristics of DEGs indicated that the DEGs had the highest expression in geneClusterC, followed by geneClusterB and geneClusterA (Figure 6(d)). We also conducted Kaplan-Meier survival analyses on the OS and PFS of patients in these three gene clusters. The results showed that geneClusterC had the highest survival rate, while geneClusterA had the lowest survival rate (Figures 6(e) and 6(f)). These findings indicated that the high expression of the m6A regulatory genes WTAP, IGFBP1, IGFBP3, and ALKBH5 might indicate a better prognosis in STS patients. In contrast, the high expression of METL3, YTHDC1, HNRNPC, LRPPRC, HNRNPA2B1, and RBMX indicated a poor prognosis for STS patients. And the high expression of DEGs indicated that these STS patients have a higher survival rate. These findings might provide beneficial help for the clinical treatment of STS.

### 3.6. Establishment of an Individualized m6A Scoring System

**Figure 7:**
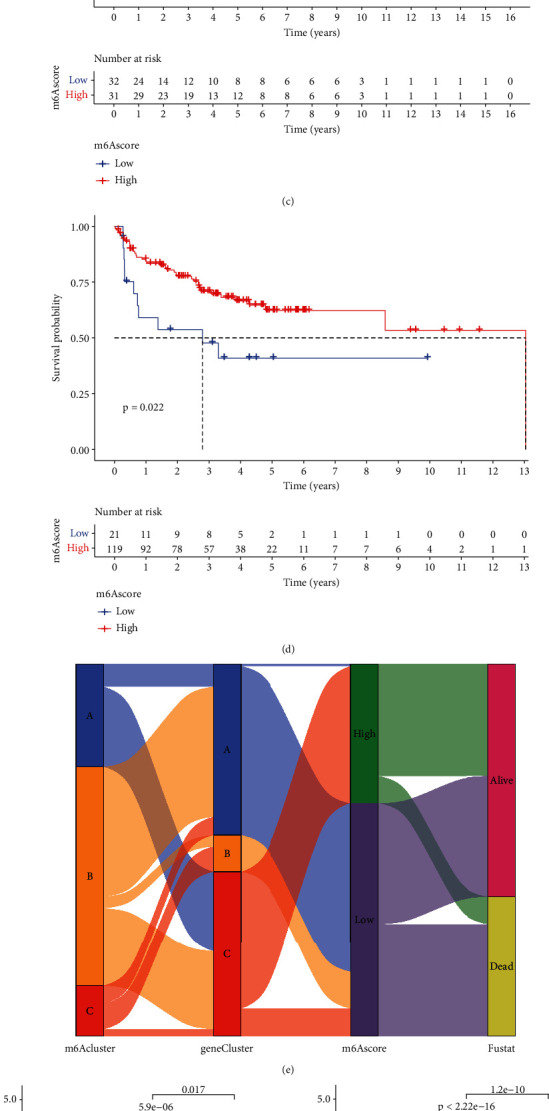
Construction of the STS m6A score. (a) Overall survival (OS) curves of STS patients with high and low m6A scores. The red curve represents the m6A high group, and the blue curve represents the m6A low group. (b) Progression-free survival (PFS) curves of STS patients with high and low m6A scores. (c) OS curves of STS patients with high and low m6A scores in the validation cohort. (d) PFS curves of STS patients with high and low m6A scores in the GEO validation cohort. (e) Sankey diagram representing the distribution of three different m6A methylation modification patterns, gene clusters, m6A scores, and survival status. (f, g) Kruskal-Wallis detection of m6A clustering and gene clustering. (h) Immune correlation analysis between m6A score and immune cell infiltration in STS (red and blue represent positive and negative correlations, respectively, and ∗ means there is a correlation between the two).

According to the above results, we demonstrated that m6A methylation modification plays a vital role in the formation and regulation of the TME in STS patients. However, the process of m6A modification in each STS patient is complex and individual. Thus, to accurately assess the individualized m6A modification pattern of each STS patient, we established an individualized m6A scoring system (m6A score) to quantify the m6A modification behavior of each STS patient. Through the above formula of the m6A score, we calculated the prognostic risk score of each STS patient and stratified them according to the score. Then, we used the “survival” R package to analyze the OS and PFS of the stratified STS patients. The results indicated that the survival rate of the high-score group was significantly different and higher compared to the low-risk group (*p* < 0.001) (Figures 7(a) and 7(b)). We also used the GSE17618, GSE17674, and GSE30929 datasets from the GEO database to verify the OS and PFS of the high and low score groups and obtained similar results (Figures 7(c) and 7(d)). To evaluate the relationship between m6A clusters, gene clusters, m6A scores, and survival, we drew a Sankey diagram (Figure 7(e)). Next, we performed a Kruskal-Wallis test on the m6A clusters, gene clusters, and m6A scores. Interestingly, significant differences were detected between m6A clusters, gene clusters, and m6A scores (*p* < 0.05). GeneClusterC had the highest median score, while GeneClusterB had the lowest. In the analysis of m6A clusters, m6A cluster C had the lowest score. The cluster with the highest score was m6A cluster A (Figures 7(f) and 7(g)). The cluster with the highest score was m6A cluster A. These results demonstrated that the m6A score is closely related to the prognosis of STS patients, and high m6A scores might indicate a good prognosis. Many studies have reported the important role of immunotherapy in malignant tumors. Therefore, to better understand the relationship between the m6A score and immune cells in STS patients, we conducted an immune correlation analysis. Activated B cells, activated dendritic cells, and gamma delta T cells were positively correlated to the m6A score (Figure 7(h)). This finding can be of great guiding significance for the immunotherapy of STS patients.

### 3.7. Evaluation of the Association between the m6A Score and Clinical Characteristics of STS Patients

**Figure 8:**
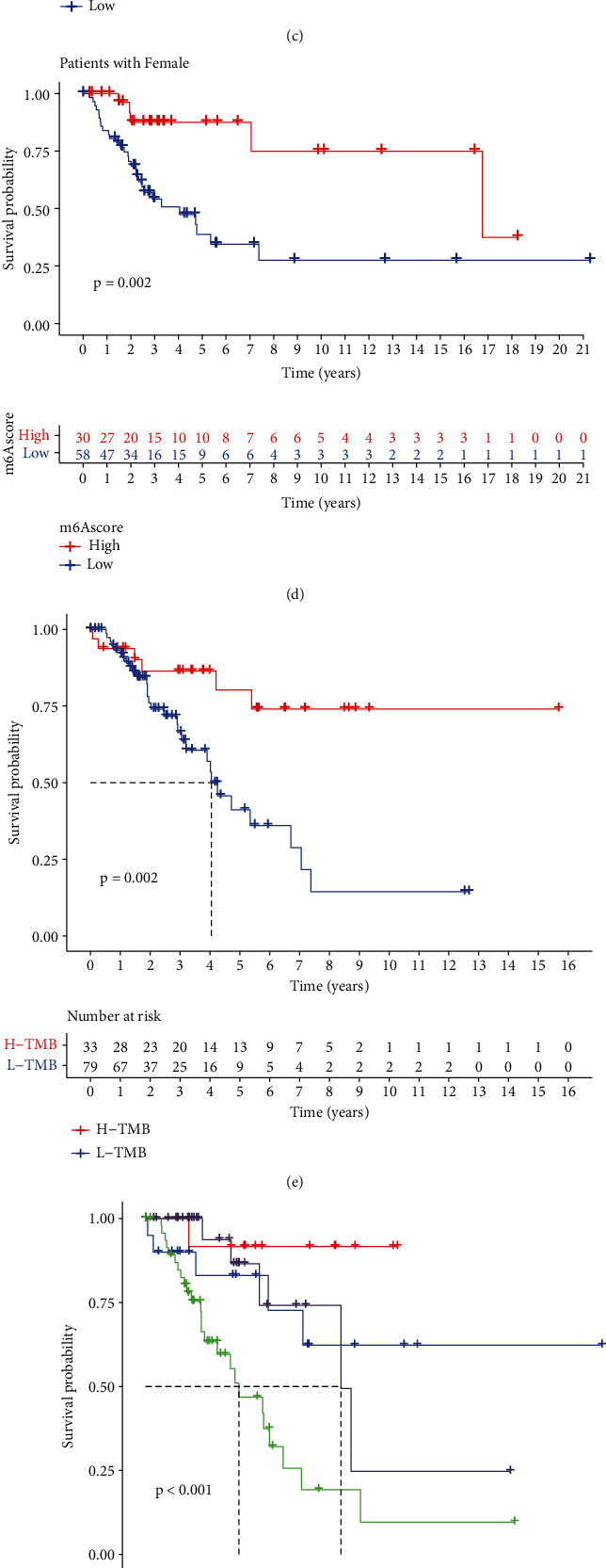
Correlation analysis between the m6A score and clinical characteristics of different STS patients. Kaplan-Meier survival analysis curve of m6A score of STS patients. (a) over 65 years. (b) Kaplan-Meier survival analysis curve of m6A score of STS patients 65 years and younger. (c) Kaplan-Meier survival analysis curve of m6A score of male STS patients. (d) Female STS patients. (e) Survival analysis of STS patients in the high and low TMB groups. (f) Survival analysis between the high and low TMB groups and high and low m6A score groups. (g, h) Tumor mutation gene waterfall chart of high and low m6A scores.

In the previous section, we analyzed the relationship between m6A scores and m6A clusters, gene clusters, and immune cells. In this section, we conducted an in-depth investigation of the relationship between the m6A score and the clinical characteristics of different STS patients. We analyzed the survival difference between the m6A score and the age and gender of STS patients. We found that among STS patients >65 years, there was no significant difference in survival between high and low m6A score patients (*p* = 0.808), while STS patients ≤65 years old have significant differences in m6A scores (*p* = 0.032), and patients with high m6A scores had a higher survival rate (Figures 8(a) and 8(b)). This shows that this m6A score system might be more suitable for the prognostic assessment of STS patients ≤65 years. We also found that regardless of whether the patient is a male or female STS patient, the m6A score had a significant correlation with it (*p* = 0.039, *p* = 0.002), and patients with a high m6A score had a better survival rate (Figures 8(c) and 8(d)), consistent with the results of our previous analysis. These results showed that the m6A scoring system can be used to assess the prognosis of STS patients of any gender.

Many studies have shown that tumor mutational burden (TMB) is inseparable from the occurrence and development of tumors. To study the relationship between TMB and m6A scores, STS patients were divided into high and low TMB groups according to the optimal cutoff, and survival analyses were carried out. The survival rate of the high TMB group was significantly higher than the low TMB group (Figure 8(e)). Moreover, in the combined survival analysis of the m6A score and TMB, the survival rate of the H-TMB+H-m6A score group was significantly higher than the other three groups (*p* < 0.001), while the survival rate of the L-TMB+L-m6A score group was the lowest (Figure 8(f)). These findings indicated that the m6A score might closely interact with TMB, and both affect the survival of STS patients. Additionally, we also plotted the tumor mutation gene waterfall chart of the high and low m6A score groups and the results showed that the overall mutation rate of the high m6A score group was 65.12% and for the low m6A score group was 71.01%. For the tenth significant mutation, the ratios of genes were 5 and 9%, respectively (Figures 8(g) and 8(h)). The TMB and m6A scores were negatively correlated. Many studies have indicated that TMB is closely related to immunotherapy, and the high state of TMB helps maintain the responsiveness of malignant tumor patients to anti-PD-1/PD-L1 immunotherapy. Herein, we showed that there is a certain correlation between TMB and m6A scores, so we speculate that the m6A modification mode of STS might play a crucial role in the clinical response of anti-PD-1/PD-L1 immunotherapy.

### 3.8. Evaluation of the Effects of the m6A Score on Anti-PD-1/L1 Immunotherapy in STS Patients

**Figure 9:**
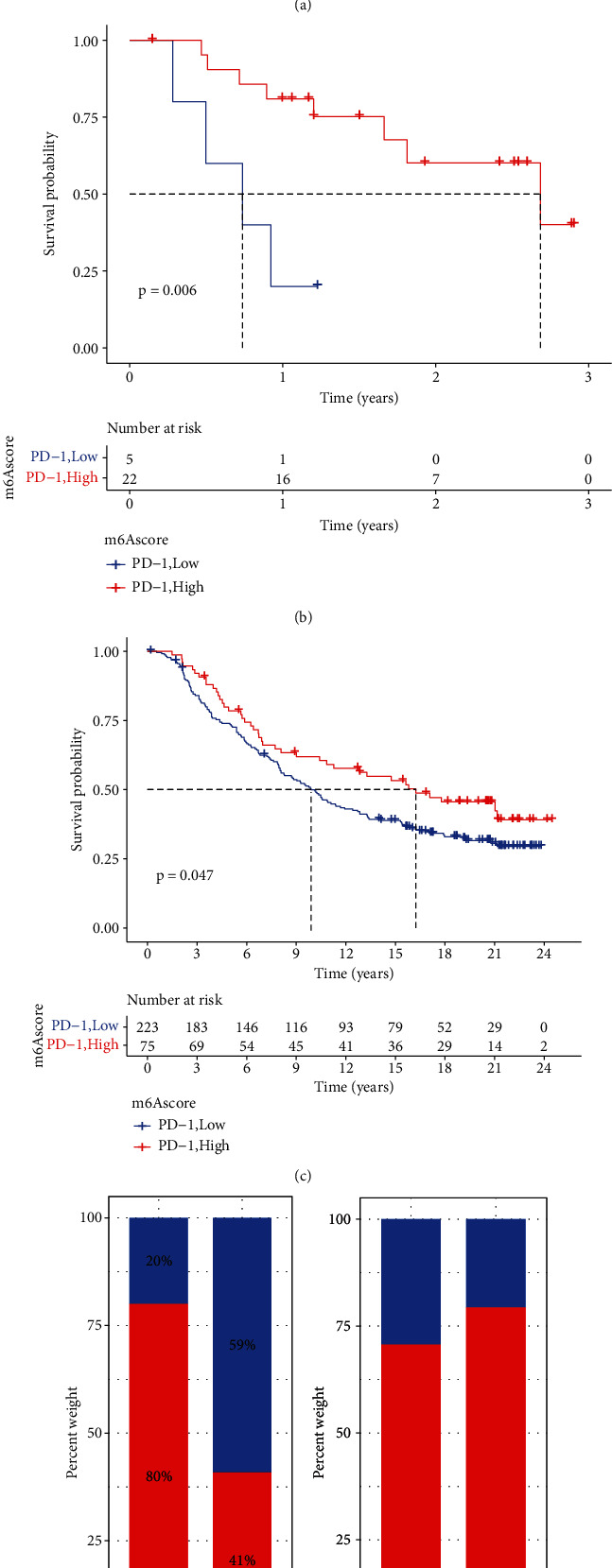
The m6A score can predict the evaluation of immunotherapy efficacy. (a) Differences in ICB-related genes in STS patients with different m6A scores (^∗^*p* < 0.05, ^∗∗^*p* < 0.01, and ^∗∗∗^*p* < 0.001). (b, c) Survival analysis curve between the high and low m6A scores in the GSE78220 and IMvigor210 cohorts receiving anti-PD1/PD-L1. (d, e) The immunotherapy response rate analysis of m6A high and low group patients in the GSE78220 and IMvigor210 cohorts to anti-PD-1 therapy (CR: complete remission; PR: partial remission; SD: stable phase; PD: disease progression).

The escape of the immune system has been identified as a sign of cancer [7]. Therefore, immunotherapy of malignant tumors is very attractive in clinical treatment. Immunotherapy has been used in the treatment of many tumors and has achieved good results. The most famous and widely used immunosuppressant is immune checkpoint blocking (ICB). Cytotoxic T lymphocyte antigen-4 (CTLA-4) and programmed cell death protein 1 (PD1/PD-L1) are the two main therapeutic approaches for ICB [32, 33]. To evaluate whether the m6A scoring system can guide the immunotherapy of STS patients, we conducted the following experiments. First, we analyzed the differences in ICB-related genes (PDCD1 and CD274) in patients with different m6A scores. The results suggested that PDCD1 and CTLA4 are significantly different between the m6A high and low groups (*p* < 0.05), and in the high group, the expression level was higher (Figure 9(a)). These results indicated that, among STS patients, patients with high m6A scores may be more responsive to anti-PD-1/PD-L1 or anti-CTLA4 immunotherapy. To verify the accuracy of the above conclusions, we downloaded an independent cohort (GSE78220, IMvigor210) receiving anti-PD-1/PD-L1 treatment from the GEO database and used the same statistical analysis method to divide them into two groups (high and low m6A), and then performed Kaplan-Meier survival analysis and immunotherapy response rate analysis. These results showed that, among patients receiving anti-PD-1/PD-L1 treatment, patients with a high m6A score had a higher OS rate than the group with low m6A scores (Figures 9(b) and 9(c)) (*p* < 0.05). Additionally, patients with high m6A scores (59% and 29%) also had a higher immune response rate than those with low scores (20% and 21%). These results were consistent with the results of the above analysis (Figures 9(d) and 9(e)).

## 4. Discussion

STS is a relatively rare heterogeneous stromal tumor and has more than 70 different histological subtypes [34]. The incidence of STS is under 0.006%, accounting for about 1-2% of all adult cancers [34, 35]. Although medicine has advanced rapidly, there are few breakthroughs in STS treatment. The mechanisms of m6A regulatory factors in tumor progression have been the focus of many studies. For example, METTL3 can promote the progression of osteosarcoma by regulating the m6A level of LEF1 [36]. In bladder cancer (BLCA), the increase in the expression level of METL3 can upregulate m6A levels of the CDCP1 gene, which promotes the proliferation, migration, and invasion of BLCA [37, 38]. The interaction between m6A modification and m6A regulatory factors plays a vital role in many fields, including antitumor, inflammation, and immunity. Most of the current research has focused on studying the role of a single m6A regulatory factor, but the occurrence, development, and metastasis of tumors include the combination of multiple m6A regulatory factors that are not yet fully elucidated. Therefore, clarifying the role of different m6A regulatory factors and m6A modification modes in TME infiltration is of great interest to the understanding of STS and to guide treatment. In the present study, we used TCGA and GSE17118 (GEO) cohort data to analyze and finally obtain three different m6A methylation modification patterns and establish an STS-related m6A scoring system. Different evaluations and verifications demonstrated the accuracy of the m6A scoring system in predicting the prognosis of STS patients, which has great potential for guiding immunotherapy and provided new ideas for distinguishing and classifying STS patients.

At present, the cause of STS has not been fully elucidated. It has been reported that the occurrence of STS is related to gene mutations. For example, patients with mutations in the RB1 tumor suppressor gene have a significantly increased incidence of STS [39]. Furthermore, structural activation of oncogenic signaling pathways caused by oncogene mutations negatively affects the TME by promoting intratumoral immune cell rejection or facilitating the recruitment of immunosuppressive cells [40]. However, the treatment of STS still lacks efficient methods. Due to the success of immunotherapy for tumors such as melanoma, prostate cancer, and renal cell carcinoma, the immunotherapy of STS has regained the attention of many scholars. Herein, the GO and KEGG enrichment analysis showed that STS-related m6A differential genes are rich in immune pathways, including neutrophil activation and participation in the immune response. The ssGSEA showed that there were also significant differences in the content of immune cells among the three m6A clusters: the content of immune cells in m6A cluster A was higher than in the other two groups. Except for CD56 bright natural killer cells, m6A cluster C had the lowest immune cell content. These findings showed that immunity plays an important role in the m6A modifications of STS. Immunotherapy may break the bottleneck of STS treatment and bring new hope for STS patients. There is much evidence that TMB can help in the diagnosis and treatment of tumors. For example, TMB can be used as an immune marker to predict the response of immunotherapy and used to select tumor patients who benefit from immune checkpoint inhibitor therapy. Studies have indicated that TMB can accurately predict the immune response of PD1/PD-1 [41]. Additionally, previous studies have shown that in stage IV or recurring non-small-cell lung cancer (NSCLC), patients with high TMB have longer periods of PD-1 blockade when receiving first-line nivolumab compared with platinum-based chemotherapy. In the present study, we found that the top five m6A regulators with the highest mutation frequency were ZC3H13, RBM15, YTHDC2, FMR1, and WTAP. Gong et al. have shown that the low expression of ZC3H13 indicates the poor prognosis of breast cancer, and its downregulation is related to the tumor progression of triple-negative breast cancer patients [42]. Previous studies have also found that ZC3H13 inhibits the Ras-ERK signaling pathway, reduces the expression of Snail, Cyclin D1, and Cyclin E1, and upregulates the expression of occludin and Zo-1, thereby inhibiting tumor progression [43]. At present, there are few reports on the mechanism of the other five m6A regulatory factors in STS; thus, more research is required. According to the results of the CNV frequency analysis, the frequency of obtaining ZC3H13 was significantly lower than the frequency of losing it. It has been reported that CNV is closely related to mRNA expression and the prognosis of sarcoma [44]. These results indicated that m6A might be closely related to the prognosis of STS patients. However, there are few reports on the mechanisms of the other five m6A regulatory factors in STS, and more research is required.

As mentioned earlier, the TME has individual heterogeneity. Thus, the treatment of tumor patients is also personalized. Therefore, we constructed an m6A scoring system that can perform individualized and quantitative evaluation of STS patients. We divided STS patients into two groups based on the m6A score and conducted a survival analysis. A significant difference in survival was detected between the high and low score groups. Next, to explore the roles of the m6A score in STS, we conducted ssGSEA and CIBERSORT immune infiltration analysis. We found that m6A scores were correlated with activated B cells, activated CD4 T cells, activated CD8 T cells, activated dendritic cells, CD56 natural killer cells, macrophages, and natural killer T cells. The expression of cells was also positively correlated. Combining the results of the m6A score survival analysis, ssGSEA, and CIBERSORT immune infiltration analysis, we concluded that the group with high m6A scores had better OS and PFS. Hence, m6A scores might have a major relationship with the activation of many immune cells. The m6A score can not only accurately assess the prognosis and survival of STS patients but can also be used for STS immunotherapy. We found that there are significant differences in ICB gene levels between the high and low m6A score groups, which are mainly manifested in the difference in the expression of CTLA4 and PDCD1. The expression levels of CTLA4 and PDCD1 in the high m6A group were higher than those in the low group (*p* < 0.05). Additionally, we verified these results using the GEO dataset (GSE78220, IMvigor210) with PD-1/PD-L1 treatment, and the results of this analysis were consistent with the above results. Therefore, the m6A score is a new method for evaluating the prognosis and survival of STS patients. It is more stable and accurate than other clinical indicators and can be used to evaluate the effect of immunotherapy and to screen the most suitable candidates for immunotherapy. It can provide clinicians with new directions and provide new ideas for clinical guidance in the treatment of STS.

Nevertheless, this study also has limitations. First, this was a retrospective study, and all analyses were based on in silico results and lacked in vitro and in vivo verification. Therefore, further prospective studies are needed to verify our results. Second, the relationship between m6A regulatory factors and STS and TME and the specific regulatory mechanisms described here also need to be verified by in vivo and in vitro experiments in the future. Briefly, we used bioinformatics and various statistical analyses to establish an STS-related m6A scoring system and explained its possible mechanisms of action. The m6A score can be used to assess the survival and prognosis of each STS patient and to predict the effects of immunotherapy, deepening the understanding of STS and providing new possibilities for clinicians to treat STS.

## 5. Conclusions

In summary, we revealed the influence of different m6A modification modes on the TME and the mechanisms of action through bioinformatics analysis and verification using three public databases (TCGA, GEO, and GTEx). The different m6A modification patterns might be one of the important factors leading to the heterogeneity and complexity of TME. Moreover, the STS-related m6A score can be used to comprehensively evaluate the individuality of STS patients, predict their survival rate and immunotherapy responsiveness, deepen our understanding of STS-related TME, cell infiltration, immunity, and gene mutations, and guide clinical treatment.

## Figures and Tables

**Table 1:** Clinical information of STS patients in TCGA database.

ID	OS time	PFS time	OS-fustat	Age	Sex	Race	Cancer type	Metastasis	Radiotherapy	Metastatic sites
TCGA-VT-A80G	0.85	0.89	Alive	66	Male	White	UPS	Yes	Yes	Lung
TCGA-QQ-A5V2	1.84	0.15	Alive	42	Male	White	UPS	NA	NA	NA
TCGA-IE-A4EJ	3.22	0.26	Alive	84	Female	White	UPS	NA	NA	NA
TCGA-SG-A6Z4	3.45	1.57	Alive	47	Male	White	UPS	Yes	Yes	Lung
TCGA-K1-A6RT	4.24	1.44	Alive	48	Male	White	LMS	NA	Yes	NA
TCGA-DX-AB2E	4.34	1.48	Alive	60	Male	White	MFS	Yes	No	Others, specify
TCGA-Z4-A8JB	4.37	0.36	Alive	24	Female	White	Other	NA	No	NA
TCGA-K1-A6RV	5.19	0.43	Alive	67	Male	White	LMS	NA	Yes	NA
TCGA-DX-A8BP	7.1	1.17	Alive	85	Male	White	UPS	Yes	No	Lung
TCGA-MB-A5Y9	8.28	0.69	Alive	90	Male	White	UPS	NA	No	NA
TCGA-QC-A7B5	8.51	1.08	Alive	75	Male	White	UPS	No	No	NA
TCGA-MJ-A68J	8.8	1.49	Alive	55	Female	Black	MFS	NA	Yes	NA
TCGA-SI-AA8C	8.97	1.63	Alive	20	Female	White	Other	Yes	Yes	Lung
TCGA-DX-AB2S	9.2	0.76	Alive	53	Female	White	MFS	No	Yes	NA
TCGA-RN-AAAQ	10.09	1.39	Alive	52	Male	White	Other	No	Yes	NA
TCGA-3B-A9HY	10.32	0.86	Alive	49	Male	White	LMS	No	No	NA
TCGA-DX-A8BG	10.84	1.49	Alive	83	Female	White	MFS	Yes	Yes	Lung
TCGA-X9-A971	10.91	2.27	Alive	52	Female	White	LMS	No	No	NA
TCGA-DX-A8BO	10.94	3.39	Alive	67	Female	White	UPS	No	No	NA
TCGA-VT-A80J	11.66	2.17	Alive	49	Female	White	UPS	Yes	No	Other, specify
TCGA-VT-AB3D	12.45	1.03	Alive	71	Male	White	UPS	No	Yes	NA
TCGA-UE-A6QT	13.21	1.1	Alive	50	Female	Asian	UPS	No	No	NA
TCGA-Z4-A9VC	13.37	1.11	Alive	37	Male	White	DLP	NA	No	NA
TCGA-UE-A6QU	13.4	1.11	Alive	90	Female	Asian	UPS	No	No	NA
TCGA-DX-A7ET	15.21	1.26	Alive	71	Male	Black	UPS	No	No	NA
TCGA-Z4-AAPF	15.93	1.32	Alive	35	Female	White	Other	NA	No	NA
TCGA-DX-AB2Q	16.1	5.55	Alive	65	Female	White	UPS	Yes	Yes	Others, specify
TCGA-SG-A849	17.51	1.45	Alive	78	Male	White	UPS	No	Yes	NA
TCGA-QC-AA9N	17.64	1.47	Alive	53	Female	Black	UPS	No	No	NA
TCGA-3B-A9HZ	17.94	3.2	Alive	66	Male	White	LMS	Yes	Yes	Lung
TCGA-X6-A7WD	18.33	2.56	Alive	63	Female	White	LMS	No	No	NA
TCGA-LI-A67I	18.86	2.52	Alive	75	Female	White	MFS	No	Yes	NA
TCGA-X6-A8C2	19.09	2.92	Alive	56	Male	White	UPS	No	Yes	NA
TCGA-X6-A8C3	19.15	1.59	Alive	59	Female	Asian	MFS	No	Yes	NA
TCGA-IE-A4EI	19.51	1.62	Alive	67	Female	White	LMS	No	No	NA
TCGA-MB-A8JL	19.71	1.64	Alive	53	Female	White	LMS	No	No	NA
TCGA-IE-A6BZ	19.88	1.65	Alive	65	Female	White	UPS	No	No	NA
TCGA-QC-A6FX	20.93	1.74	Alive	68	Male	White	UPS	No	Yes	NA
TCGA-MB-A8JK	21.71	1.8	Alive	49	Male	White	UPS	No	Yes	NA
TCGA-DX-A8BT	21.94	3.36	Alive	63	Female	White	UPS	No	Yes	NA
TCGA-MJ-A850	22.37	1.86	Alive	28	Male	White	Other	No	Yes	NA
TCGA-MB-A5Y8	25.39	2.11	Alive	60	Female	White	UPS	NA	Yes	NA
TCGA-DX-AB2T	25.76	6.48	Alive	54	Female	Black	MFS	Yes	No	Lung
TCGA-3B-A9HI	25.99	4.16	Alive	68	Male	White	DLP	Yes	Yes	Lung
TCGA-X6-A8C7	29.43	2.45	Alive	24	Female	White	Other	No	Yes	NA
TCGA-SI-A71Q	32.72	2.72	Alive	34	Female	Black	Other	No	Yes	NA
TCGA-X6-A7WC	33.8	3.77	Alive	74	Male	White	LMS	No	Yes	NA
TCGA-DX-A6YU	34.33	2.86	Alive	50	Female	White	MFS	No	No	NA
TCGA-X6-A7WA	35.87	2.98	Alive	90	Female	White	LMS	No	No	NA
TCGA-QQ-A5VC	35.87	2.98	Alive	63	Female	White	LMS	No	No	NA
TCGA-3B-A9HJ	36.27	3.02	Alive	68	Male	White	DLP	No	Yes	NA
TCGA-QQ-A5VD	37.09	3.09	Alive	52	Male	White	LMS	No	No	NA
TCGA-DX-A6B9	37.39	3.11	Alive	45	Female	Black	LMS	No	No	NA
TCGA-DX-A8BN	38.14	3.17	Alive	78	Female	White	UPS	No	Yes	NA
TCGA-DX-A6YR	40.97	3.41	Alive	75	Male	White	MFS	No	Yes	NA
TCGA-DX-A3LT	42.81	4.08	Alive	62	Male	White	DLP	NA	Yes	NA
TCGA-DX-A8BL	45.11	3.75	Alive	59	Male	White	UPS	No	No	NA
TCGA-DX-A48K	45.96	3.83	Alive	65	Male	White	LMS	NA	No	NA
TCGA-DX-A8BM	47.83	3.98	Alive	60	Male	White	UPS	No	Yes	NA
TCGA-DX-AB2Z	50.36	4.19	Alive	87	Female	White	UPS	No	No	NA
TCGA-DX-AB3B	50.85	4.23	Alive	28	Female	White	Other	No	No	NA
TCGA-DX-A8BQ	51.87	4.32	Alive	63	Male	White	UPS	No	Yes	NA
TCGA-IF-A4AK	52.23	4.35	Alive	82	Female	White	LMS	No	Yes	NA
TCGA-K1-A3PO	53.29	4.44	Alive	42	Male	White	LMS	No	NA	NA
TCGA-DX-AB3C	61.01	5.93	Alive	27	Male	White	Other	Yes	Yes	Lung
TCGA-DX-A8BK	61.93	5.16	Alive	61	Female	White	UPS	No	Yes	NA
TCGA-DX-AB2L	65.93	5.49	Alive	35	Male	White	MFS	No	No	NA
TCGA-DX-A6YT	67.15	5.59	Alive	31	Female	White	MFS	No	No	NA
TCGA-HB-A2OT	67.58	5.63	Alive	78	Female	Black	UPS	No	NA	NA
TCGA-DX-A6YS	78.22	6.51	Alive	61	Male	White	MFS	No	Yes	NA
TCGA-DX-AB2O	86.04	7.17	Alive	78	Female	White	UPS	No	No	NA
TCGA-DX-A6YZ	86.24	7.18	Alive	59	Male	White	MFS	No	Yes	NA
TCGA-QQ-A8VB	88.11	15.66	Alive	68	Female	White	Other	No	No	NA
TCGA-DX-AB32	101.91	8.49	Alive	51	Male	White	MFS	No	Yes	NA
TCGA-DX-AB2W	106.37	8.86	Alive	62	Female	White	UPS	No	Yes	NA
TCGA-DX-AB2V	106.47	8.87	Alive	81	Male	White	MFS	No	Yes	NA
TCGA-QQ-A8VF	111.89	9.32	Alive	70	Male	White	LMS	No	No	NA
TCGA-DX-A8BR	150.23	12.51	Alive	63	Female	White	UPS	No	Yes	NA
TCGA-DX-A7EO	152	12.66	Alive	20	Female	White	Other	No	Yes	NA
TCGA-DX-A6B8	1.38	1.32	Dead	80	Male	White	LMS	Yes	Yes	Lung
TCGA-DX-A8BZ	2.96	0.54	Dead	78	Female	Black	LMS	Yes	No	Lung
TCGA-K1-A6RU	4.66	1.94	Dead	66	Female	White	MFS	NA	Yes	NA
TCGA-X6-A7W8	4.8	2.2	Dead	89	Male	White	MFS	No	Yes	NA
TCGA-DX-AB2P	5.29	1	Dead	79	Male	White	UPS	Yes	Yes	Lung
TCGA-DX-AB2X	7.16	1.1	Dead	73	Female	White	UPS	Yes	No	Lung
TCGA-DX-A6YX	7.29	1.4	Dead	68	Female	NA	MFS	Yes	Yes	Lung
TCGA-DX-A3UE	7.36	2.9	Dead	66	Female	White	LMS	NA	Yes	NA
TCGA-DX-A8BH	8.94	1.2	Dead	86	Male	White	UPS	No	No	NA
TCGA-DX-A48L	10.38	2.02	Dead	49	Female	White	LMS	NA	Yes	NA
TCGA-QQ-A5V9	13.3	3.89	Dead	76	Male	White	UPS	No	No	NA
TCGA-LI-A9QH	13.99	1.47	Dead	72	Female	White	UPS	No	Yes	NA
TCGA-WK-A8Y0	15.8	1.88	Dead	49	Female	White	UPS	Yes	Yes	Lung
TCGA-X6-A8C4	16.56	1.71	Dead	70	Female	White	UPS	No	Yes	NA
TCGA-DX-AB30	16.56	1.9	Dead	53	Female	White	MFS	Yes	Yes	Lung
TCGA-DX-A7EF	16.79	1.77	Dead	88	Female	White	UPS	Yes	Yes	Lung
TCGA-DX-A6YV	16.95	4.2	Dead	74	Male	White	MFS	Yes	No	Other, specify
TCGA-SI-A71O	22.44	1.9	Dead	29	Male	White	Other	Yes	Yes	Lung
TCGA-DX-A7EQ	24.38	2.45	Dead	72	Male	White	Other	Yes	Yes	Lung
TCGA-DX-A23U	28.68	6.36	Dead	81	Male	White	DLP	NA	NA	NA
TCGA-X6-A8C6	32.03	2.92	Dead	55	Male	White	MFS	No	Yes	NA
TCGA-DX-A8BU	34	3.05	Dead	58	Male	White	UPS	Yes	Yes	Lung
TCGA-WK-A8XZ	36.2	4.71	Dead	56	Female	White	LMS	Yes	Yes	NA
TCGA-DX-A3U7	42.18	4.24	Dead	67	Male	White	LMS	NA	No	NA
TCGA-QQ-A5VB	48.49	4.04	Dead	53	Female	White	LMS	Yes	No	Other, specify
TCGA-QQ-A8VG	60.84	5.39	Dead	52	Male	White	Other	No	No	NA
TCGA-3B-A9HR	64.85	7.37	Dead	38	Female	Black	LMS	Yes	Yes	Lung
TCGA-IE-A3OV	79.99	6.7	Dead	42	Male	White	LMS	Yes	NA	Lung

**Table 2:** Clinical information of patients on the GSE17118 dataset.

ID	OS time	OS-fustat	Age	Sex	Grade	Histology
GSM428182	Months: 36	Alive	Age: 65	Sex: F	1	Myxoid
GSM428190	Months: 123	Alive	Age: 55	Sex: F	1	Myxoid
GSM428193	Months: 120	Alive	Age: 34	Sex: F	1	Myxoid
GSM428196	Months: 222	Alive	Age: 57	Sex: F	1	WD, lipoma-like
GSM428203	Months: 105	Alive	Age: 54	Sex: M	1	Dedifferentiated
GSM428185	Months: 182	Dead	Age: 68	Sex: M	1	Dedifferentiated
GSM428200	Months: 58	Dead	Age: 60	Sex: F	1	Dedifferentiated
GSM428160	Months: 26	Alive	Age: 66	Sex: F	2	Epithelial solid
GSM428161	Months: 25	Alive	Age: 37	Sex: F	2	Epithelial solid
GSM428175	Months: 30	Alive	Age: 29	Sex: M	2	Epithelial solid
GSM428180	Months: 110	Alive	Age: 46	Sex: M	2	WD, lipoma-like
GSM428181	Months: 32	Alive	Age: 43	Sex: M	2	Myxoid-round cell
GSM428186	Months: 200	Alive	Age: 54	Sex: F	2	Myxoid-round cell
GSM428189	Months: 57	Alive	Age: 48	Sex: F	2	Myxoid-round cell
GSM428192	Months: 201	Alive	Age: 56	Sex: M	2	Myxoid-round cell
GSM428194	Months: 65	Alive	Age: 47	Sex: M	2	Myxoid-round cell
GSM428195	Months: 21	Alive	Age: 62	Sex: M	2	WD
GSM428201	Months: 34	Alive	Age: 66	Sex: F	2	Pleomorphic
GSM428202	Months: 34	Alive	Age: 66	Sex: F	2	Pleomorphic
GSM428206	Months: 59	Alive	Age: 51	Sex: M	2	Malignant peripheral nerve sheath tumor
GSM428214	Months: 45	Alive	Age: 33	Sex: F	2	Malignant peripheral nerve sheath tumor
GSM428179	Months: 27	Dead	Age: 52	Sex: F	2	Epithelial tubulopapillary
GSM428197	Months: 25	Dead	Age: 74	Sex: M	2	Dedifferentiated
GSM428198	Months: 204	Dead	Age: 73	Sex: F	2	Myxoid-round cell
GSM428199	Months: 30	Dead	Age: 42	Sex: F	2	Myxoid-round cell
GSM428217	Months: 13	Dead	Age: 28	Sex: F	2	Malignant peripheral nerve sheath tumor
GSM428218	Months: 31	Dead	Age: 28	Sex: F	2	Malignant peripheral nerve sheath tumor
GSM428219	Months: 5	Dead	Age: 28	Sex: F	2	Malignant peripheral nerve sheath tumor
GSM428177	Months: 90	Alive	Age: 47	Sex: M	3	Epithelial tubulopapillary
GSM428178	Months: 34	Alive	Age: 52	Sex: F	3	Epithelial tubulopapillary
GSM428187	Months: 259	Alive	Age: 75	Sex: F	3	Dedifferentiated
GSM428207	Months: 82	Alive	Age: 44	Sex: M	3	Malignant peripheral nerve sheath tumor
GSM428162	Months: 40	Dead	Age: 66	Sex: M	3	Epithelial solid
GSM428163	Months: 6	Dead	Age: 42	Sex: F	3	Epithelial solid
GSM428164	Months: 36	Dead	Age: 55	Sex: M	3	Epithelial solid
GSM428165	Months: 24	Dead	Age: 58	Sex: F	3	Epithelial solid
GSM428166	Months: 27	Dead	Age: 73	Sex: F	3	Epithelial solid
GSM428169	Months: 33	Dead	Age: 63	Sex: M	3	Biphasic
GSM428184	Months: 94	Dead	Age: 84	Sex: M	3	Pleomorphic
GSM428191	Months: 40	Dead	Age: 51	Sex: F	3	Pleomorphic
GSM428204	Months: 9	Dead	Age: 64	Sex: F	3	Malignant peripheral nerve sheath tumor
GSM428209	Months: 52	Dead	Age: 20	Sex: M	3	Malignant peripheral nerve sheath tumor
GSM428212	Months: 7	Dead	Age: 59	Sex: M	3	Malignant peripheral nerve sheath tumor
GSM428213	Months: 10	Dead	Age: 59	Sex: F	3	Malignant peripheral nerve sheath tumor
GSM428215	Months: 19	Dead	Age: 42	Sex: M	3	Malignant peripheral nerve sheath tumor
GSM428216	Months: 8	Dead	Age: 24	Sex: F	3	Malignant peripheral nerve sheath tumor
GSM428170	Months: 32	Alive	Age: 22	Sex: M	Unknown	Epithelial
GSM428171	Months: 14	Alive	Age: 54	Sex: M	Unknown	Epithelial
GSM428172	Months: 31	Alive	Age: 58	Sex: M	Unknown	Epithelial
GSM428174	Months: 22	Alive	Age: 52	Sex: M	Unknown	Epithelial
GSM428168	Months: 10	Dead	Age: 65	Sex: F	Unknown	Biphasic
GSM428173	Months: 9	Dead	Age: 74	Sex: M	Unknown	Epithelial
GSM428176	Months: 10	Dead	Age: 53	Sex: F	Unknown	Biphasic
GSM428188	Months: 240	Dead	Age: 64	Sex: F	Unknown	Myxoid-round cell
GSM428205	Months: 31	Dead	Age: 26	Sex: F	Unknown	Malignant peripheral nerve sheath tumor
GSM428210	Months: 30	Dead	Age: 28	Sex: M	Unknown	Malignant peripheral nerve sheath tumor
GSM428211	Months: 31	Dead	Age: 23	Sex: M	Unknown	Malignant peripheral nerve sheath tumor

**Table 3:** The 23 m6A regulators associated with the prognosis of soft tissue sarcoma patients were identified by Cox and KM analyses.

ID	HR	HR.95L	HR.95H	*p* value	km
METTL3	0.925695	0.552877	1.549912	0.769054	0.202209
METTL14	0.946089	0.393809	2.27289	0.901374	0.083591
METTL16	1.630622	1.050971	2.529972	0.029125	0.000902
WTAP	0.71246	0.401332	1.264785	0.246953	0.016138
VIRMA	2.20093	1.121481	4.319373	0.021834	0.024044
ZC3H13	0.525344	0.31021	0.889677	0.016626	7.45*E* − 06
RBM15	0.988187	0.615363	1.586891	0.960782	0.076116
RBM15B	1.320175	0.738591	2.359712	0.348562	0.065019
YTHDC1	0.693648	0.350715	1.371907	0.293149	0.033798
YTHDC2	0.972287	0.514104	1.838817	0.931116	0.324887
YTHDF1	1.507948	0.702825	3.235381	0.291623	0.072694
YTHDF2	2.417459	1.118277	5.225994	0.024821	0.011167
YTHDF3	1.408248	0.859169	2.308234	0.174496	0.024574
HNRNPC	1.405069	0.706132	2.795821	0.332656	0.010345
FMR1	1.293923	0.824723	2.030058	0.262139	0.014454
LRPPRC	1.306818	0.731777	2.333736	0.365749	0.076072
HNRNPA2B1	1.11485	0.64813	1.917656	0.694414	0.046348
IGFBP1	1.025577	0.824382	1.275875	0.820679	0.27507
IGFBP2	1.085802	0.932566	1.264218	0.288904	0.020057
IGFBP3	0.999422	0.807793	1.236511	0.995756	0.011666
RBMX	1.076471	0.637519	1.817654	0.782782	0.149265
FTO	1.2204	0.692466	2.150827	0.490885	0.013611
ALKBH5	1.045958	0.735977	1.486499	0.802157	0.001157

## Data Availability

The 120 STS-related sample data and the GSE17118 dataset used to support the findings of this study have been deposited in the TCGA repository (https://portal.gdc.cancer.gov/) and the GEO database (https://www.ncbi.nlm.nih.gov/geo/), respectively.

## Abbreviations

TME: Tumor microenvironment
STS: Soft tissue sarcoma
CNV: Copy number variation
GEO: Gene Expression Omnibus
TCGA: The Cancer Genome Atlas
UCSC: University of California Santa Cruz
GSVA: Gene set variation analysis
ssGSEA: Single-sample gene set enrichment analysis
PCA: Principal component analysis
m6A: N6-methyladenosine
DEGs: Differentially expressed genes.
